# Blood-feeding Patterns of Amazonian Forest Edge Mosquitoes

**DOI:** 10.21203/rs.3.rs-9717303/v1

**Published:** 2026-06-11

**Authors:** Victoria Bernardi, Livia Sacchetto, Beatriz Marques, Cecilia Banho, Leonardo da Rocha, Adam Hendy, Nelson Fe, Michaela Buenemann, Maria Paula Mourao, Marcus Lacerda, Kathryn Hanley, Nikos Vasilakis, Mauricio Nogueira

**Affiliations:** Faculdade de Medicina de São José do Rio Preto; Faculdade de Medicina de São José do Rio Preto; Faculdade de Medicina de São José do Rio Preto; Faculdade de Medicina de São José do Rio Preto; Faculdade de Medicina de São José do Rio Preto; The University of Texas Medical Branch at Galveston; Fundação de Medicina Tropical; New Mexico State University; Fundação de Medicina Tropical; Fundação de Medicina Tropical; New Mexico State University; The University of Texas Medical Branch at Galveston; Faculdade de Medicina de São José do Rio Preto

**Keywords:** Amazon rainforest, forest edge environments, mosquito blood meals, engorged mosquitoes, arbovirus vectors

## Abstract

**Background:**

Mosquitoes are key arbovirus vectors and understanding their blood-feeding habits is crucial to elucidate potential pathogen transmission dynamics. Forest edge habitats, where natural and human-modified environments intersect, can enhance interactions among humans, wildlife, and mosquitoes, potentially enabling viral spillover and spillback. Characterizing these interactions is essential for assessing public health risks associated with diseases and guiding targeted vector management strategies.

**Methods:**

Female mosquitoes were collected across forest edge habitats in the Adolpho Ducke forest reserve, Manaus, Brazil, using hand nets, between May 2021 and June 2022. Specimens were morphologically identified, and observable blood-engorged females were selected for blood meal analysis. DNA was extracted, and the mitochondrial cytochrome c oxidase subunit I (COI) gene was amplified and sequenced using next-generation sequencing (NGS).

**Results:**

A total of 141 engorged mosquitoes were selected for blood-feeding analysis, from which COI sequences were successfully obtained in 111 (78.7%) specimens. Psorophora amazonica was the predominant species (82.9%), followed by Ps. albigenu (6.3%), Haemagogus janthinomys (4.5%), and Culex coronator (1.8%). Other species, including Aedes albopictus, Hg. baresi, Ps. albipes, Ps. circumflava, and Sabethes batesi, were each represented by a single individual (0.9%). Human DNA was identified in most blood meals (88.3%), while non-human DNA included ungulates (6.3%), rodents (1.8%), non-human primates (1.8%), birds (0.9%) and carnivores (0.9%). The distribution of sampling sites showed that anthropophilic and zoophilic interactions occurred simultaneously in the same landscapes, reflecting the overlap of wild and anthropogenic habitats.

**Conclusions:**

Anthropophilic mosquitoes inhabiting the edges of Amazonian forests exhibited opportunistic blood-feeding behavior, predominantly taking blood from humans but occasionally feeding on a broad diversity of non-human vertebrate hosts. This pattern is likely driven by the spatial contiguity between wild and urban/agricultural environments, which facilitates vector-mediated contact among multiple vertebrate species. These findings highlight the epidemiological relevance of forest edge habitats and reinforce the need for continuous entomological surveillance and targeted vector control strategies to mitigate the risk of arbovirus spillover and spillback.

## Background

Mosquitoes (family *Culicidae*) are of major epidemiological importance, acting as vectors for a wide range of pathogens that affect both humans and animals, especially in tropical regions such as Brazil [1]. Their vector competence is intrinsically linked to the obligate hematophagy of females, a behavior essential for egg development which can enable the acquisition and transmission of infectious agents [2]. Mosquitoes select vertebrate hosts for blood meals based not only on species-specific traits but also on ecological factors, such as microclimate, habitat fragmentation, and host availability. Feeding patterns, in turn, affect disease transmission and opportunities for pathogens to spill over among different host species [3–5].

Among mosquito-transmitted pathogens, arboviruses (arthropod-borne viruses) represent a significant public health challenge in tropical regions, where ecological conditions favor their maintenance and dissemination [6]. The Amazon ecosystem, with its high vertebrate biodiversity and abundance of mosquito species constitutes a particularly complex scenario for arbovirus transmission [7–9]. The epidemiology of these viruses is largely shaped by the natural dynamics of vector-host interactions, which determine the maintenance and amplification of viral cycles in the environment [10]. However, the recent intensification of exploitation of natural areas may reconfigure these interactions, influencing feeding patterns and the risk of pathogen exchange between wildlife and human populations [7,11–13].

In this context, edges between forests and urban or agricultural land covers emerge as interfaces where the coexistence of high biodiversity and high human occupancy creates conditions favorable for viral spillover and spillback events. Mosquitoes, as well as humans and other vertebrate hosts, can move between these environments. Such cross-environmental mobility increases the likelihood of contact with mosquitoes that may function as bridge vectors, thereby linking sylvatic and urban transmission cycles and promoting the emergence or reemergence of arboviruses [11,12,14,15].

The municipality of Manaus, located in the state of Amazonas, Brazil, provides a unique setting for investigating mosquito-host interactions. Situated in a transition zone, the city combines intense urbanization with the proximity of continuous forest areas, creating ideal conditions for interactions between natural and human-modified environments [11]. Furthermore, the simultaneous circulation of multiple arboviruses, combined with the high abundance and diversity of mosquito vectors in the region, creates a favorable setting for investigating potential transmission cycles and assessing local epidemiological risks [11,16,17]

Despite recognition that ecological interfaces constitute hotspots for arbovirus transmission, gaps remain in characterization of mosquito blood-feeding patterns along the Amazonian forest edge [11,12,14,15]. Therefore, the present study aims to characterize mosquito feeding patterns in forest edge areas of Manaus. Understanding these patterns can provide data-driven insights to guide public health interventions in regions facing complex ecological and anthropogenic challenges.

## Methods

### Ethical Considerations

Field collections of mosquitoes differed from human landing collections in that mosquitoes were captured using nets prior to landing and no skin was deliberately exposed to attract mosquitoes. All collectors were vaccinated against yellow fever virus (YFV) and wore trousers and either a long-sleeved shirt or repellent to minimize the risk of being bitten. Mosquito collections were in accordance with Brazilian environmental regulations and were authorized by the appropriate governmental agency under SISBIO permit number 57003–6, issued on September 3, 2020.

### Mosquito Sampling and Processing

**Figure 1:**
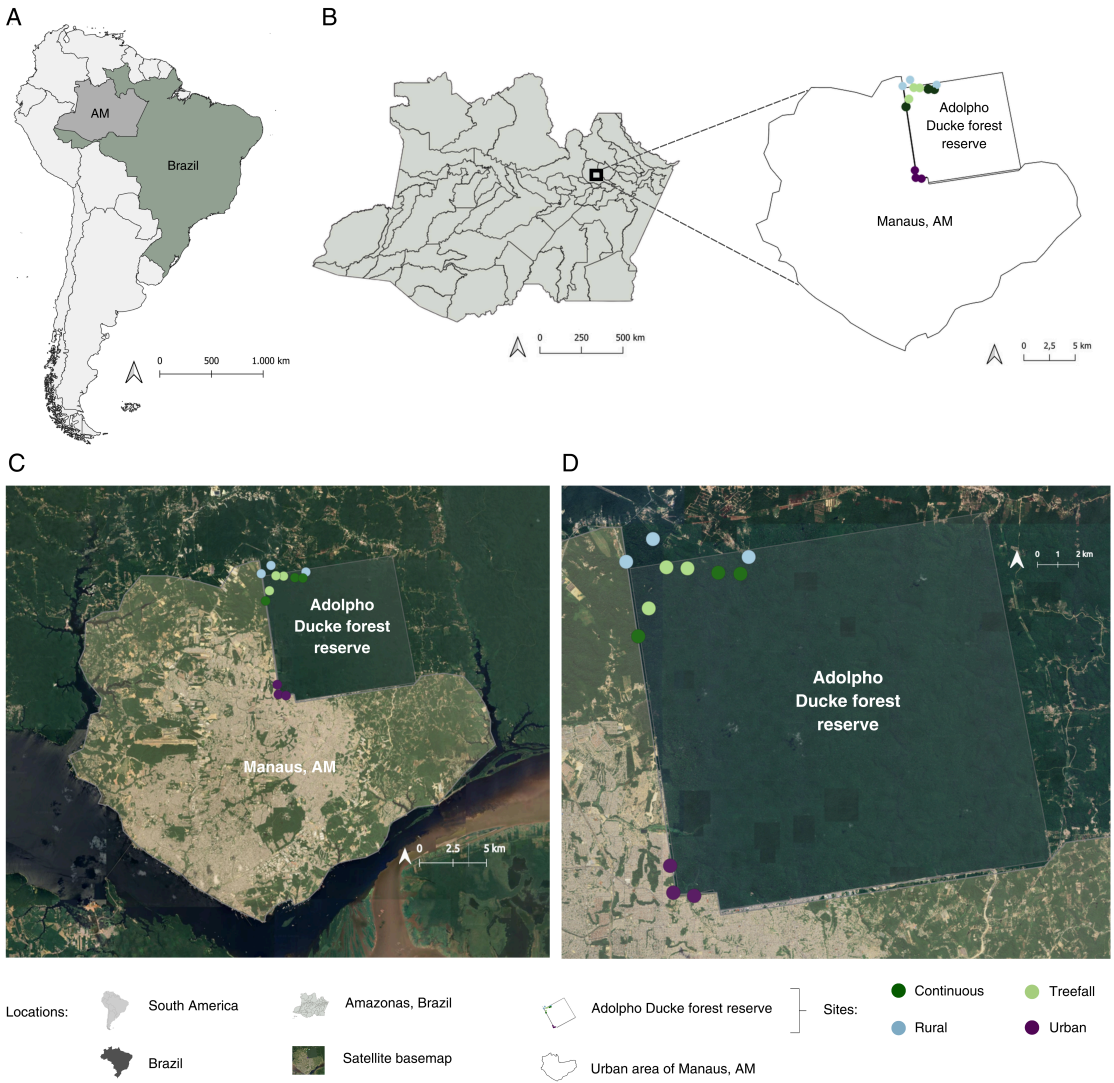
Study area. (A) Geographic location of the state of Amazonas (AM) in Brazil. (B) The map highlights the state of Amazonas and the location of Manaus. An enlarged detail provides a view of the urban area of Manaus and the location of the Adolpho Ducke forest reserve. (C) Satellite image showing the location of the Adolpho Ducke forest reserve in relation to the urban area of Manaus and the distribution of mosquito sampling sites along the forest edge. Sampling points are categorized according to environmental context: continuous forest (dark green), treefall gaps (light green), rural fragments (light blue), and urban fragments (purple). (D) Detailed view of the Adolpho Ducke forest reserve showing the spatial distribution of sampling sites within and around the reserve according to the same environmental categories. An interactive HTML map of the study area and sampling sites is available in the data repository (Mendeley Data: https://data.mendeley.com/datasets/4zkb3v8rt3/2). Some graphic elements were created using Canva Pro: https://www.canva.com/design/DAG9p7mBXZM/XSftCr59deLjQypVDAIlUg/view?utm_content=DAG9p7mBXZM&utm_campaign=designshare&utm_medium=link2&utm_source=uniquelinks&utlId=hd974722384. Shapefiles were obtained from the Brazilian government through IBGE on its official website, available at: https://www.ibge.gov.br/geociencias/organizacao-do-territorio/malhas-territoriais/15774-malhas.html

Between May 2021 and June 2022, mosquito specimens were collected in the Adolpho Ducke forest reserve, a 100 km^2^ area of primary forest located in Manaus, Amazonas, Brazil. Three biologically independent sites were established within each environmental category: continuous forest, treefall gaps, urban forest edges, and rural forest edges. Urban and rural edge sites were located along forest borders adjacent to residential or agricultural areas, whereas treefall gap sites were positioned within natural canopy openings caused by fallen trees, and continuous forest sites were located in closed-canopy forest areas serving as controls. Thus, the study comprised 12 sampling sites in total, each separated by at least 500 m to minimize spatial autocorrelation. Urban and rural edge sites were established in locations considered safe and accessible and with agreement from local communities (Fig 1A–C). Active collections of anthropophilic mosquitoes by individuals deploying hand nets were conducted during peak biting activity (10:00 to 15:00 hours), both at ground level and on platforms five meters above the ground [11].

Collected specimens were transferred to 50 mL conical tubes at 30-minute intervals. Tubes containing live mosquitoes were kept in insulated Styrofoam boxes, protected from directsunlight, and transported daily to the Dr. Heitor Vieira Dourado Tropical Medicine Foundation (FMT-HVD) for storage at −80°C. Subsequently, mosquitoes were processed on a refrigerated table (BioQuip, Rancho Dominguez, CA, USA), identified using a stereomicroscope and appropriate taxonomic keys [18–24], and preserved at −80°C until further steps.

The samples were sent to the Laboratório de Pesquisas em Virologia (LPV), Faculdade de Medicina de São José do Rio Preto (FAMERP) for molecular analysis, where they were maintained at −80°C until processing. Each sample was homogenized in 0.5 mL of 1X phosphate-buffered saline (PBS) using 5 mm steel beads with a mechanical disruptor, the L-BEADER 6 (Loccus, Cotia, SP, BR), operated for three cycles of 30 seconds each at 3,000 rpm. Homogenized samples were then centrifuged at 10,000 rpm for 10 minutes, and the resulting supernatants were carefully transferred to new 1.5 mL microtubes.

### Molecular Workflow

Genomic DNA from the homogenized samples was extracted using a magnetic bead-based protocol described by Possebon and coauthors [25]. Amplification of the mitochondrial cytochrome c oxidase subunit I (COI) gene was performed using two primer sets, yielding an amplicon of approximately 663 base pairs (bp), as described by Townzen and coauthors [26] (Table S1). PCR amplification was carried out following the Illumina Microbial Amplicon Prep (iMAP) protocol (Illumina, San Diego, CA, USA), with minor modifications. Specifically, the annealing temperature was optimized to 52 °C to improve amplification efficiency for the COI primers used in this study. Thermal cycling parameters consisted of an initial denaturation at 98 °C for 3 minutes, followed by 40 cycles of 98 °C for 15 seconds and 52 °C for 2 minutes (combined annealing/extension step).

Amplicon libraries were prepared following the Illumina Microbial Amplicon Prep (iMAP) protocol (Illumina, San Diego, CA, USA), according to the manufacturer’s instructions. Equimolar amounts of individual libraries were pooled, and the concentration and fragment size of the pooled library were assessed using a Qubit 3.0 Fluorometer (Thermo Fisher Scientific, Waltham, MA, USA) and the Agilent 4150 TapeStation system (Agilent Technologies, Santa Clara, CA, USA). Sequencing was performed on an Illumina MiSeq platform (Illumina, San Diego, CA, USA) using a MiSeq Reagent Kit v2 with a paired-end configuration (2 × 150 bp). A negative control was included throughout all steps to monitor potential contamination.

### Blood meal source identification

To perform the host identification analyses, a custom reference database of mitochondrial COI gene sequences was constructed using sequences from mammals, amphibians, reptiles, and birds obtained from the Barcode of Life Data System (BOLD) (https://boldsystems.org/) and the NCBI (National Center for Biotechnology Information) (https://www.ncbi.nlm.nih.gov/) repositories. Redundant sequences were removed from the database to minimize bias during alignment, using the Seqtk toolkit (version 1.3-r106) [27].

Raw sequencing reads were subjected to quality control using Trimmomatic (version 0.39)[28], which removed low-quality bases (minimum Phred score of Q30), sequencing adapters, primer sequences, and short reads (<50 nucleotides). Filtered reads were initially screened against the curated database using Minimap2 (version 5.0.17–1) [29]. Taxa with the highest number of mapped sequences were selected as the reference for the *map-to-reference* approach in Geneious (version 2024.0) (https://www.geneious.com/) in order to generate consensus sequences (Table S2). Species-level assignment was validated by NCBI BLASTn (https://www.ncbi.nlm.nih.gov/) and Barcode ID-BOLD (https://boldsystems.org/), retaining only sequences with ≥ 95% for host identification (Table S3). In cases where sequences showed equivalent identity to multiple species within the same genus, identification was restricted to genus level.

Data visualization was conducted in R Studio (version 3.6.3). Maps were created using QGIS Desktop (version 3.26.3), with shapefiles obtained from the Brazilian government through IBGE on its official website [30].

## Results

A total of 141 visibly engorged mosquitoes were identified and subjected to blood meal analysis, comprising *Psorophora amazonica* (117/141, 83%), *Haemagogus janthinomys* (8/141, 5.7%), *Ps. albigenu* (8/141, 5.7%), *Culex coronator* (2/141, 1.4%), *Aedes albopictus* (1/141, 0.7%), *Hg. baresi* (1/141, 0.7%), *Ps. albipes* (1/141, 0.7%), *Ps. circumflava* (1/141, 0.7%), *Sabethes batesi* (1/141, 0.7%), and *Sa. chloropterus* (1/141, 0.7%). Most engorged mosquitoes were collected from treefall gaps (76/141, 53.9%), followed by continuous forest (42/141, 29.8%), rural fragments (18/141, 12.8%), and urban fragments (5/141, 3.5%) (Table S3).

**Figure 2:**
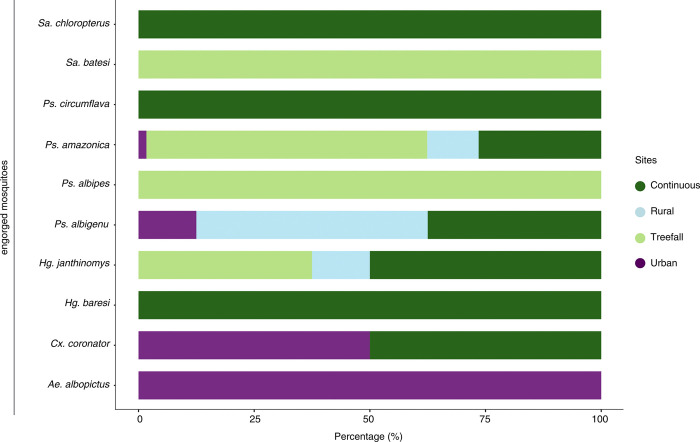
Percentage distribution of engorged mosquito species across sampling sites. Horizontal stacked bars show the relative proportion of engorged female mosquitoes collected in four habitat types: continuous forest, rural fragments, treefall gaps, and urban fragments. Percentages represent the proportion of individuals of each species found in each habitat type. *Ae. = Aedes albopictus*, *Cx.* = *Culex coronator*, *Hg.* = *Haemagogus* spp. (*Hg. baresi*, *Hg. janthinomys*), *Ps.* = *Psorophora* spp. (*Ps. albigenu*, *Ps. albipes*, *Ps. amazonica*, *Ps. circumflava*), and *Sa.* = *Sabethes*spp. (*Sa. batesi*). Some graphic elements were created using Canva Pro: https://www.canva.com/design/DAHDeZhYTzM/uLjDty7R6gCwOg10ee1D4w/view?utm_content=DAHDeZhYTzM&utm_campaign=designshare&utm_medium=link2&utm_source=uniquelinks&utlId=h72ced5218c

*Ps. amazonica*, the most abundant species, was detected in all habitat types, predominating in treefall gaps (71/117, 60.7%) and continuous forest (31/117, 26.5%), with fewer specimens collected in rural (13/117, 11.1%) and urban fragments (2/117, 1.7%). *Hg. janthinomys* occurred mainly in forested environments, including continuous forest (4/8, 50%) and treefall gaps (3/8, 37.5%), with a single specimen collected in a rural fragment (1/8, 12.5%). In contrast, *Ps. albigenu* was primarily associated with rural fragments (4/8, 50%) and continuous forest (3/8, 37.5%), with a single specimen also recorded in an urban fragment (1/8, 12.5%). The remaining species were represented by one or two individuals each: *Cx. coronator* was detected in continuous forest and urban fragments (1 each), *Ps. albipes* and *Sa. batesi* were recorded in treefall gaps, *Ps. circumflava*, *Hg. baresi*, and *Sa. chloropterus* were found in continuous forest, and *Ae. albopictus* was detected exclusively in an urban fragment (Fig 2, Table S3).

Mitochondrial COI sequences were successfully recovered from 111 of the 141 analyzed individuals (78.7%). Sequencing of these samples allowed the identification of the blood meal sources, with *Ps. amazonica* accounting for the highest number of sequences (92/111, 82.9%), followed by *Ps. albigenu* (7/111, 6.3%), *Hg. janthinomys* (5/111, 4.5%), *Cx. coronator* (2/111, 1.8%), and single successful identifications for *Ae. albopictus*, *Hg. baresi*, *Ps. albipes*, *Ps. circumflava*, and *Sa. batesi* (1/111, 0.9% each). Recovered vertebrate COI sequences obtained from mosquito blood meals showed high similarity to reference sequences, with most presenting >95% identity, ensuring reliable taxonomic assignment (Table S3).

**Figure 3:**
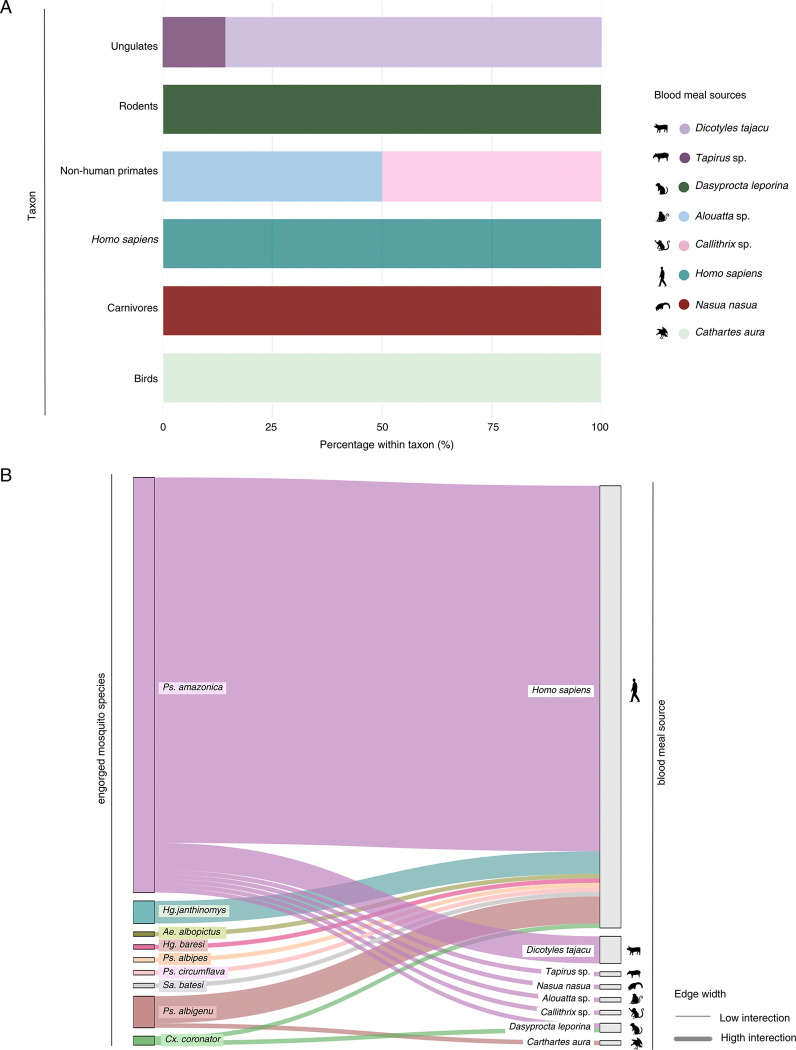
Mosquito Blood Meal Source Identification. (A) Percentage of blood meals identified by taxon. (B) Sankey diagram illustrating the interactions between mosquito species and their vertebrate blood meal sources based on mitochondrial cytochrome c oxidase subunit I (COI) gene, where the width of each link is proportional to the frequency of interaction between mosquito species and host taxa. *Ae. = Aedes albopictus*, *Cx.* = *Culex coronator*, *Hg.* = *Haemagogus* spp. (*Hg. baresi*, *Hg. janthinomys*), *Ps.* = *Psorophora* spp. (*Ps. albigenu*, *Ps. albipes*, *Ps. amazonica*, *Ps. circumflava*), and *Sa.* = *Sabethes* spp. (*Sa. batesi*). Vertebrate hosts include humans (*Homo sapiens*), ungulates (*Dicotyles tajacu*, *Tapirus* sp.), carnivores (*Nasua nasua*), NHPs (*Alouatta*sp., *Callithrix* sp.), rodents (*Dasyprocta leporina*), and birds (*Cathartes aura*). Some graphic elements were created using Canva Pro: https://www.canva.com/design/DAG9jrOiBfM/2If5iWtDfgUO1LV0Pd0vw/view?utm_content=DAG9jrOiBfM&utm_campaign=designshare&utm_medium=link2&utm_source=uniquelinks&utlId=h1b6752276b

Analysis of blood meal sources revealed a predominance of human DNA, detected in 98/111 (88.3%) specimens, indicating widespread anthropophilic feeding among the analyzed mosquitoes (Fig 2A). Among non-human sources, ungulates were the most frequent (7/111, 6.3%), with collared peccary (*Dicotyles tajacu*) as the predominant species (6/7, 85.7%), followed by tapir (*Tapirus* sp.) (1/7, 14.3%). Rodents accounted for 1.8% (2/111), with both samples being from red-rumped agouti (*Dasyprocta leporina*) (2/2, 100%). Non-human primates (NHPs) also represented 1.8% (2/111), with howler monkeys (*Alouatta* sp.) and marmosets (*Callithrix* sp.) each contributing one sample (1/2, 50% each). Carnivores accounted for 0.9% (1/111), with a single sample from the coati (*Nasua nasua*) (1/1, 100%). Birds were represented exclusively by the turkey vulture (*Cathartes aura*), which comprised the only avian blood meal detected (1/111, 0.9%) (Fig 3A, Table S3).

As previously noted, *Ps. amazonica* was the most abundant engorged species analyzed. Network analysis integrating mosquito species and identified hosts revealed distinct feeding patterns, with *Ps. amazonica* exhibiting wide dietary plasticity, with blood meals derived from humans (*Homo sapiens*) (81/92, 88%), collared peccary (*Dicotyles tajacu*) (6/92, 6.5%), tapir (*Tapirus* sp.) (1/92, 1.1%), red-rumped agouti (*Dasyprocta leporina)* (1/92, 1.1%), coati (*Nasua nasua*) (1/92, 1.1%), and NHPs (howler monkeys (*Alouatta* sp.) and marmosets (*Callithrix* sp.) (each 1/92, 1.1%)). In contrast, congeneric species, such as *Ps. albigenu*, *Ps. albipes*, and *Ps. circumflava* were found to feed predominantly on humans, although there is insufficient sampling of these species to draw conclusions about host range. A similar pattern was observed for the remaining analyzed species (Fig 3B, Table S3).

**Figure 4:**
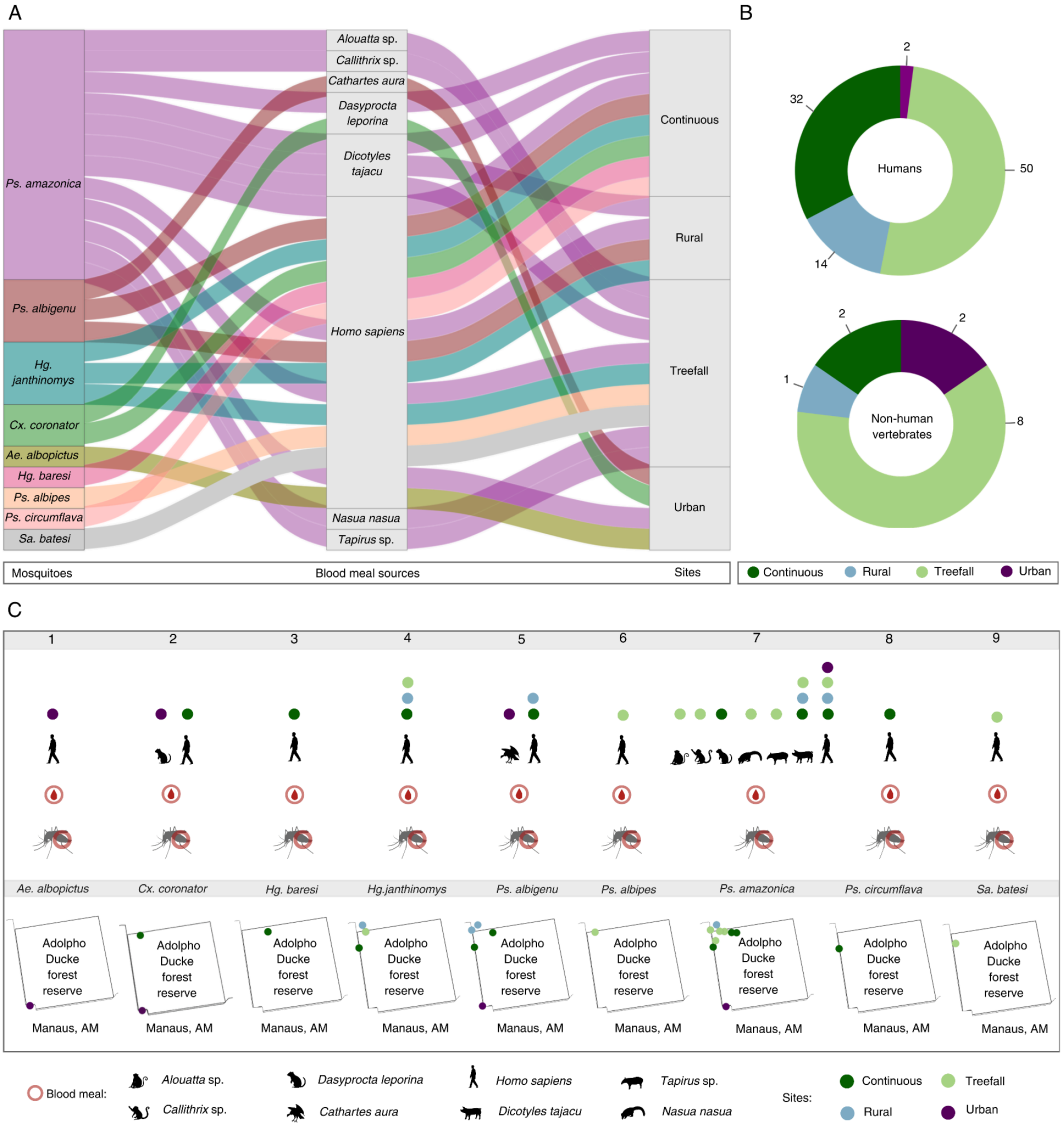
Blood Meal Source Identification in Collection Sites at the Adolpho Ducke forest reserve, Manaus, AM. (A) Sankey diagram showing interactions between mosquito species (mosquitoes), blood meal sources (blood meal), and sampling environments (sites). Each line represents a single blood-feeding event per host. Mosquito species are color coded as in the figure. (B) Donut charts illustrating the blood meals derived from humans and non-human vertebrates across different environments. Icons represent the respective vertebrate host categories (humans or non-human vertebrates). (C) Distribution of mosquito species collected at each sampling site (numbered 1 to 9), with respective blood meal sources detected. Sampling environments are indicated by colored circles: continuous forest (dark green), rural fragments (light blue), treefall gaps (light green), and urban fragments (purple). Icons show mosquito species (with genus abbreviations as above) and associated vertebrate hosts. The size of the icons represents a single blood-feeding event per host, with no repetitions. *Ae. = Aedes albopictus*, *Cx.* = *Culex coronator*, *Hg.* = *Haemagogus*spp. (*Hg. baresi*, *Hg. janthinomys*), *Ps.* = *Psorophora*spp. (*Ps. albigenu*, *Ps. albipes*, *Ps. amazonica*, *Ps. circumflava*), and *Sa.* = *Sabethes* spp. (*Sa. batesi*). Some graphic elements were created using Canva Pro: https://www.canva.com/design/DAG9jueU1GE/DHwzNJhH9gAcWiRfTA0_4w/view?utm_content=DAG9jueU1GE&utm_campaign=designshare&utm_medium=link2&utm_source=uniquelinks&utlId=hcb4ba692b4

Based on the geographic distribution of sampling points, blood-feeding interactions were evaluated across different environmental contexts. Unexpectedly, human bloodmeals were predominantly taken in treefall gaps (50/98, 51%) and continuous forest (32/98, 32.7%), rather than in rural fragments (14/98, 14.3%) and urban fragments (2/98, 2%). Among the human bloodmeals recorded in treefall gaps, 45/50 (90%) were taken by *Ps. amazonica*, followed by *Hg. janthinomys w*ith 3/50 (6%), and single records for *Ps. albipes* and *Sa. batesi* (1/50, 2% each). In the continuous forest, of the 32 human bloodmeals observed, 25/32 (78.1%) were taken by *Ps. amazonica*, 3/32 (9.4%) by *Ps. albigenu*, and single bloodmeals were taken by *Hg. baresi*, *Hg. janthinomys*, and *Ps. circumflava* (1/32, 3.1% each). In rural fragments, 10/14 (71.4%) human bloodmeals were taken by *Ps. amazonica*, 3/14 (21.4%) by *Ps. albigenu*, and 1/14 (7.1%) by *Hg. janthinomys*. In urban fragments, 1/2 (50%) human bloodmeals were taken by *Ae. albopictus* and 1/2 (50%) by *Ps. amazonica* (Fig 4A–C, Table S3).

In contrast, blood meals from non-human vertebrates (13/111, 11.7%) were primarily detected in treefall gaps (8/13, 61.5%), with occasional records in continuous forest (2/13, 15.4%), urban fragments (2/13, 15.4%), and rural fragments (1/13, 7.7%). The bloodmeals from non-human vertebrate hosts in treefall gaps (8/8, 100%) were taken by *Ps. amazonica*. Similarly, in continuous forest, all feeding events (2/2, 100%) on non-human vertebrate hosts were also carried out by *Ps. amazonica.* In urban fragments, an equal division was observed, with 50% (1/2) of the bloodmeals from non-human vertebrate hosts taken by *Cx. coronator* and the other 50% (1/2) by *Ps. albigenu*. In rural fragments, 100% of the single blood meal from a non-human vertebrate host (1/1) was taken by *Ps. amazonica.* (Fig 4A–C, Table S3).

## Discussion

Understanding mosquito blood-feeding patterns is essential for elucidating the ecological complexity of vector-host interactions that directly influence arbovirus transmission dynamics. Transitional environments, such as forest edges where sylvatic and anthropogenic habitats overlap, can facilitate interspecific pathogen transmission [10,11,15,31]. In this context, the present study provides new insights into mosquito blood-feeding patterns in forest edge areas of the Amazon.

Engorged, anthropophilic female mosquitoes were collected in the Adolpho Ducke forest reserve, including at the urban-forest interface of Manaus, Brazil. Specimens included species from the genera *Aedes*, *Culex*, *Haemagogus*, *Psorophora*, and *Sabethes*, several of which are recognized as important public health arbovirus vectors that transmit major diseases such as dengue, Zika, chikungunya, and yellow fever [32–35]. The presence of these vectors in this zone underscores the potential for arbovirus transmission, including their role as bridge vectors connecting sylvatic and urban cycles.

Based on our analyses, *Ps. amazonica* was the most abundant engorged species. This species is notable for occupying a wide range of forest strata, from the ground to the canopy, reflecting its behavioral flexibility and inherent capacity for interactions with different vertebrate hosts [11,15,36]. Our results support this pattern, showing that *Ps. amazonica* exhibits generalist feeding behavior, evidenced by blood meals from humans and a broad range of wild vertebrates. Despite its aggressive behavior, the species-specific role of *Ps. amazonica* in arbovirus transmission remains largely uncharacterized. However, previous studies have considered members of this genus as potential arbovirus vectors [37–39]. In this context, it is important to highlight the detection of blood meals from NHPs. These hosts are highly susceptible to arboviruses and play a central role in the maintenance of sylvatic transmission cycles, particularly for YFV, with which they have been historically associated and for which high lethality has been [40–42]. Thus, these findings demonstrate the interaction of *Ps. amazonica* with important vertebrate hosts involved in the maintenance of arboviruses, highlighting the need for targeted experimental studies to better understand its vector competence and epidemiological relevance.

In contrast, species of the genera *Haemagogus* and *Sabethes* are well established as primary vectors in the sylvatic YFV cycle, showing a strong preference for NHP hosts [32,43,44]. However, in our analyses, individuals from both genera (*Hg. baresi*, *Hg. janthinomys* and *Sa. batesi*) exhibited anthropophilic feeding, a behavior previously observed in related species [45,46]. According to previous studies, *Haemagogus* and *Sabethes* species are primarily canopy-dwelling [46–48]. The detection of human blood meals in this study demonstrates vector-human contact at the ecological transition areas, likely reflecting the influence of environmental changes on host availability and vertical distribution [11,44,48]. Notably, YFV has recently been detected in female *Haemagogus* mosquitoes from this reserve, highlighting the ongoing risk of viral reemergence and dissemination [16]. The convergence of sylvatic vector anthropophily and documented viral circulation poses a persistent risk of viral spillover into the human population. Although the region is endemic and outbreaks have been mitigated by high vaccination coverage, a decline in immunization rates was reported in 2023 [49]. This scenario with the reported viral circulation [16] underscores the need to strengthen vaccination campaigns and enhance entomological surveillance.

Additionally, the detection of blood-fed females of *Ae. albopictus* and *Cx. coronator* provides epidemiologically relevant evidence. Despite the limited sample size, the identification of a human blood meal in *Ae. albopictus* confirms the interaction of this species with human hosts in the study area. *Ae. albopictus*, widely distributed across the Amazon [11], is recognized as a secondary dengue virus (DENV) vector [35] and has recently been found naturally infected with Zika virus (ZIKV) in the Manaus region [17]. In contrast, in the present study, engorged female *Cx. coronator* mosquitoes fed on both humans and wild rodents (*Dasyprocta leporina*). Species of the genus *Culex* are recognized for their ability to exploit natural and human-modified environments [50], including species that are potential vectors of arboviruses in Brazil [9,34]. Blood-feeding on wild rodents is particularly relevant, as these animals are frequently described as hosts and reservoirs of several zoonotic viruses, including arboviruses, and exhibit opportunistic ecological behavior, which may favor viral circulation across different environments [9,51]. These feeding patterns are consistent with observations from other studies reporting similar behavior among *Aedes* and *Culex* populations across different ecological settings [52–55].

As expected, based on our collection method (hand nets), we detected a predominance of human derived blood meals, although feeding on a variety of non-human vertebrates was also observed. The geographic distribution of sampling sites indicated an overlap between natural and anthropogenic environments, suggesting that humans and other vertebrates share the same ecological space, thereby creating opportunities for interactions between mosquitoes and multiple vertebrate hosts. Interestingly, a higher proportion of human blood meals occurred in forested landscapes. These results indicate that, despite the diversity of available vertebrate hosts, humans are frequently encountered and fed upon, a pattern also observed by Young and coauthors [56]. These behavioral shifts may facilitate the maintenance of arbovirus transmission cycles and elevate the risk of both spillover and spillback events [10,13,57–59].

These findings reinforce the importance of understanding vector feeding patterns and monitoring the interface zones between wild and anthropogenic environments. Such areas, often characterized by intense ecological dynamics and high human pressure, are critical for entomological surveillance and for elucidating the ecological determinants of arbovirus transmission [10,13,57,59]. The Amazon illustrates such complex ecological settings, with its remarkable biodiversity and the tight spatial interface between humans and wildlife, as demonstrated in this study.

Some limitations of this study should be acknowledged. The use of hand nets for mosquito collection was appropriate for the overall project objectives and allowed the capture of several species of blood fed females for this study. Nevertheless, hand nets are heavily biased towards the collection of human-host-seeking mosquitoes, therefore, the use of alternative methods for sampling host seeking mosquitoes, such as CDC light traps [60] and BG-Sentinel traps [61], might have resulted in a different composition of blood sources, but these traps rarely entice engorged mosquitoes. Alternatively, methods for sampling resting mosquitoes would have provided an unbiased representation of blood-feeding patterns [62], but pilot studies of these methods failed to yield mosquitoes in our hands in this setting. Furthermore, the ingested blood source could not be identified for some of the samples because blood digestion hindered the recovery of DNA suitable for molecular analysis, a limitation also reported in similar studies [26,55,63,64]. Finally, for some vertebrate species such as *Alouatta*, *Callithrix*, and *Tapirus*, species-level identification could not be confidently achieved due to similar identity scores among closely related species.

## Conclusion

Overall, this study demonstrates that anthropophilic mosquitoes inhabiting forest edge environments in the Amazon exhibit opportunistic hematophagy, with a predominance of blood meals from humans and occasional feedings on wildlife. This pattern suggests that the spatial overlap between natural and anthropogenic environments increases host availability and promotes interactions between mosquitoes and multiple vertebrate species. The coexistence of anthropophilic and zoophilic feeding within the same landscapes underscores the epidemiological importance of forest edge habitats, as these settings may facilitate both arbovirus spillover and spillback. These results reinforce the need for continuous entomological surveillance and targeted investigations in forest edge areas, particularly in regions like the Amazon, which, in addition to its high biodiversity, is also recognized as a hotspot for arboviruses.

## Supplementary Material

This is a list of supplementary files associated with this preprint. Click to download.

• Supplementarytable.xlsx

• floatimage1.png

## Data Availability

Consensus sequences have been deposited in the NCBI GenBank database under accession numbers (PZ148398-PZ148508). All other study data are available in the Mendeley repository (https://data.mendeley.com/datasets/4zkb3v8rt3/2).

## Abbreviations

AM: Amazonas
Ae: Aedes
COI: mitochondrial cytochrome c oxidase subunit I
Cx: Culex
DENV: dengue virus
DNA: deoxyribonucleic Acid
Hg: Haemagogus
NGS: next generation sequencing
Ps: Psorophora
Sa: Sabethes
YFV: yellow fever virus
ZIKV: Zika virus
